# Supplementary material for: Balancing nutrient remobilization and photosynthesis: proteomic insights into the dual role of lupin cotyledons after germination
Source: Plant J. 2025 Jul 21;123(2):e70357. doi: 10.1111/tpj.70357 (PMC12279399; doi:10.1111/tpj.70357)
Supplement: Supplementary file 1 — Figure S1. Scheme of the experimental set‐up. Figure S2. Protein abundance profiles of functional protein categories during Lupinus albus cotyledon development. Figure S3. Free amino acid contents of cotyledons and true leaves at day 12. Figure S4. Carbohydrate and lipid contents in cotyledons. Figure S5. Mineral contents of shoots and roots at day 28. Figure S6. Nitrogen saving strategies after removal of the cotyledons. [file TPJ-123-0-s005.pdf]

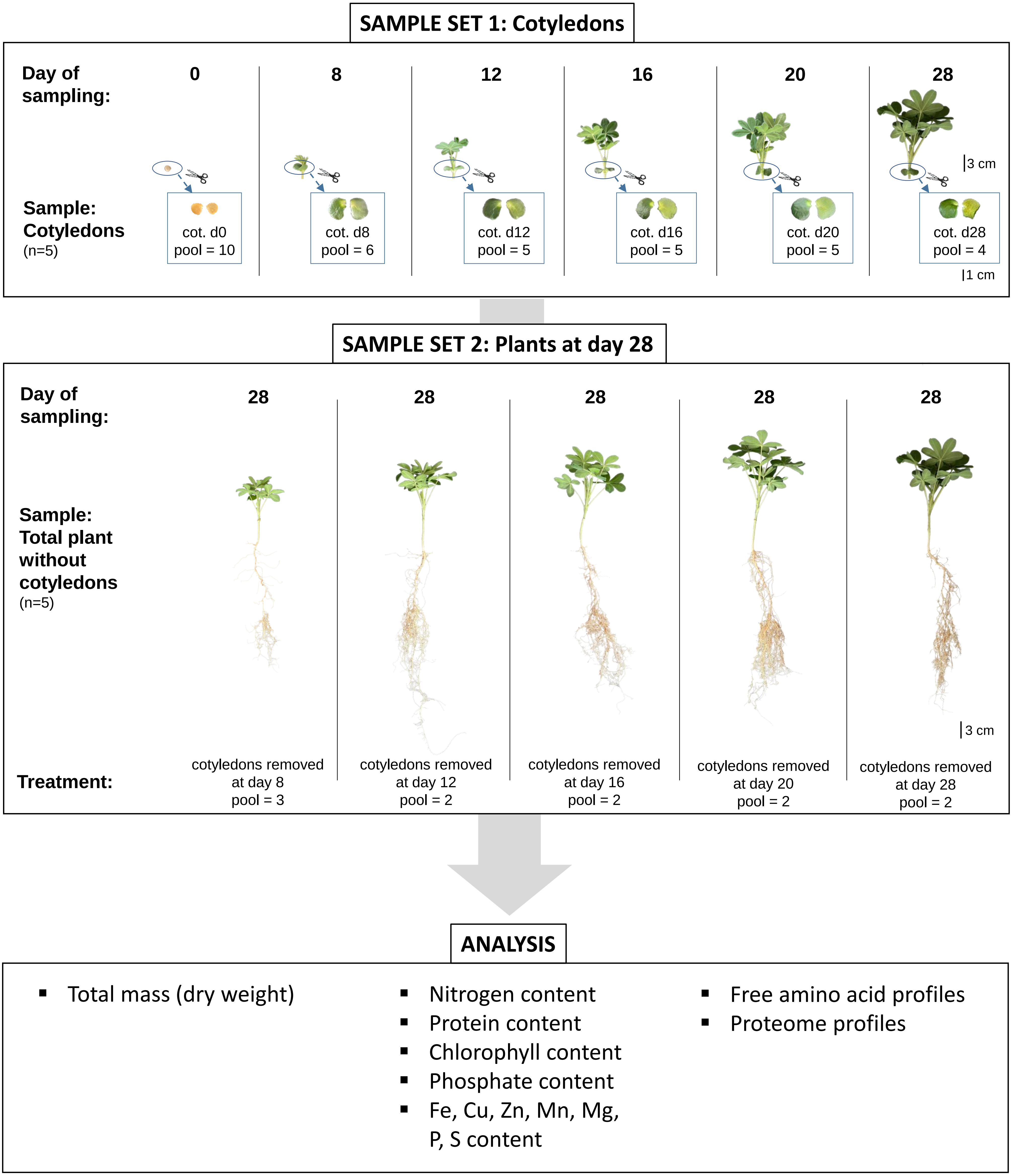

Supplementary Figure S1: Scheme of the experimental set-up: *Lupinus albus* plants were grown for twenty-eight days the absence of rhizobia and without nitrogen fertilization. The cotyledons were removed from subsets of these plants at day 8, 12, 16, 20 or 28 after sowing and harvested for subsequent analysis. Cotyledons of seeds were harvested as control. The remaining plants without cotyledons were harvested for subsequent analysis at day 28. The complete dataset is provided as Supplementary datasets S1 and S2.

A

Functional protein categories	Number of proteins	Protein [mg · g DW⁻¹] 0d	8d	12d	16d	20d	28d
Storage proteins	23	93.7	45.7	25.4	12.8	9.5	11.4
Protein metabolism	1005	47.0	42.1	40.5	36.2	34.0	31.3
Lipid metabolism	220	46.9	19.5	16.4	11.8	9.7	8.9
Vesicle trafficking	134	1.6	1.2	1.4	1.3	1.1	1.2
Cell	323	33.7	12.2	11.4	9.2	8.0	8.6
Stress	169	15.5	9.3	9.1	7.7	7.9	8.9
Redox	131	11.2	8.9	9.1	8.1	7.6	7.9
Secondary metabolism	115	2.5	2.4	2.4	2.4	2.1	1.8
Amino acid metabolism	167	2.3	5.3	5.4	5.1	4.7	4.1
Carbohydrate metabolism	270	11.9	12.7	13.4	12.6	11.3	10.8
Cellular respiration	200	9.5	16.2	18.0	16.6	15.1	14.3
Coenzyme metabolism	111	1.0	2.6	2.2	2.0	1.8	1.8
Nucleotide metabolism	73	1.2	1.6	1.8	1.7	1.7	1.7
Photosynthesis	191	3.1	45.1	58.9	62.9	62.3	62.2
Signalling	79	0.9	1.2	1.5	1.5	1.5	1.8
DNA	64	2.0	2.2	2.9	3.3	3.8	4.2
RNA	313	2.8	3.1	3.3	3.2	3.3	3.2
Nutrient uptake	19	0.2	0.3	0.3	0.2	0.3	0.4
Hormone metabolism	73	1.6	1.2	1.6	1.5	1.5	2.0
Transport	180	7.0	6.7	6.8	6.7	6.6	7.1

B

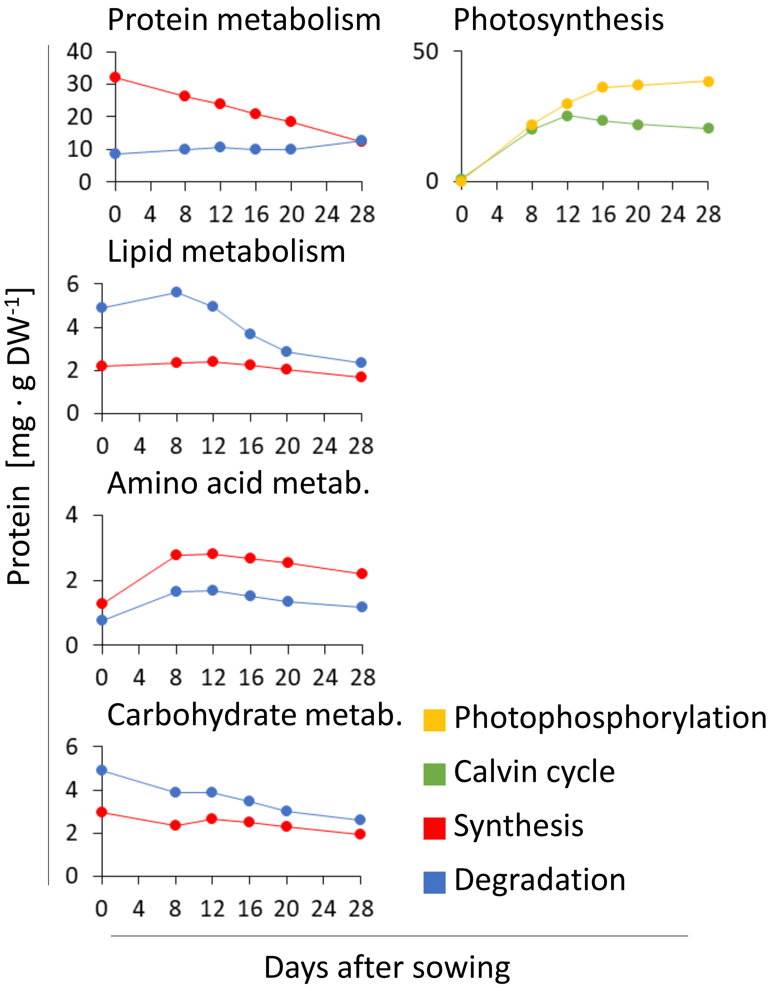

Supplementary Figure S2: Protein abundance profiles of functional protein categories during *Lupinus albus* cotyledon development. (**A**) Relative protein abundance of functional protein categories and number of different proteins detected in the respective category. Categories were adapted from MapMan4. (**B**) Protein abundance profiles of selected functional categories. The complete dataset is provided as Supplementary dataset S2.

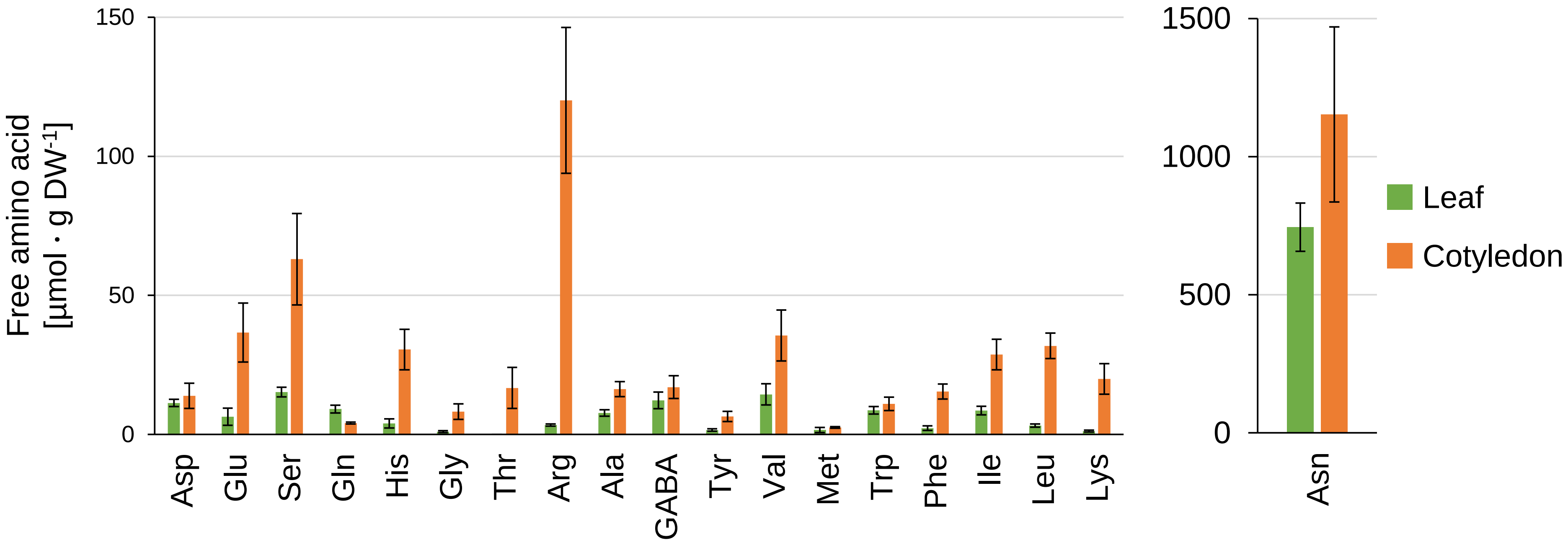

Supplementary Figure S3: Free amino acid contents of cotyledons and true leaves at day 12. The complete dataset is provided as Supplementary dataset S3.

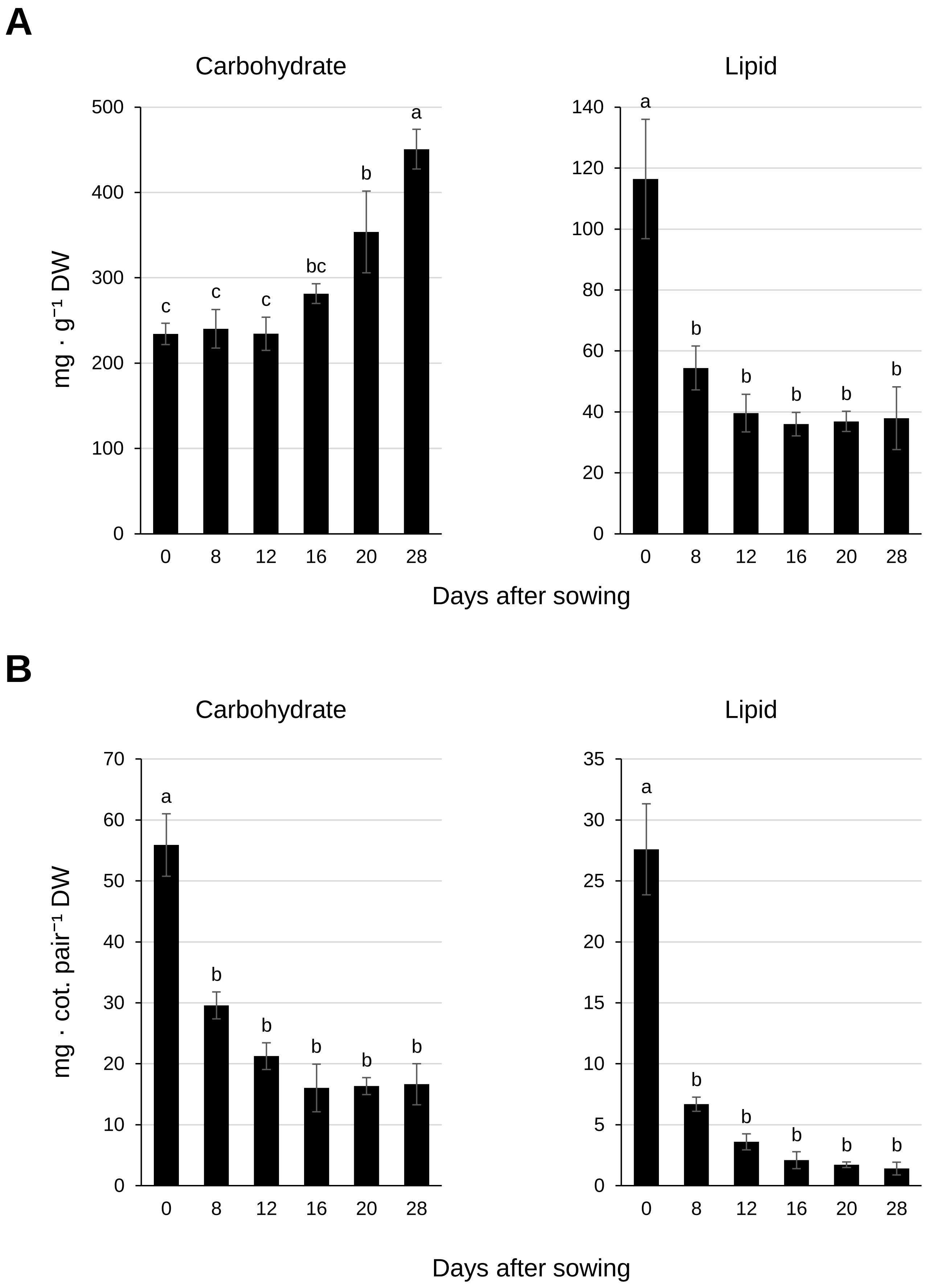

Supplementary Figure S4: Carbohydrate and lipid quantities in cotyledons. (**A**) Relative amounts. (**B**) Absolute amounts. Data presented are means ± SD (n = 5). Means were compared using one-way ANOVA followed by Tukey's HSD test. Letter-based significance grouping was used at a significance level of $p < 0.05$; means sharing the same letter are not significantly different. Complete dataset is provided as Supplementary dataset S1.

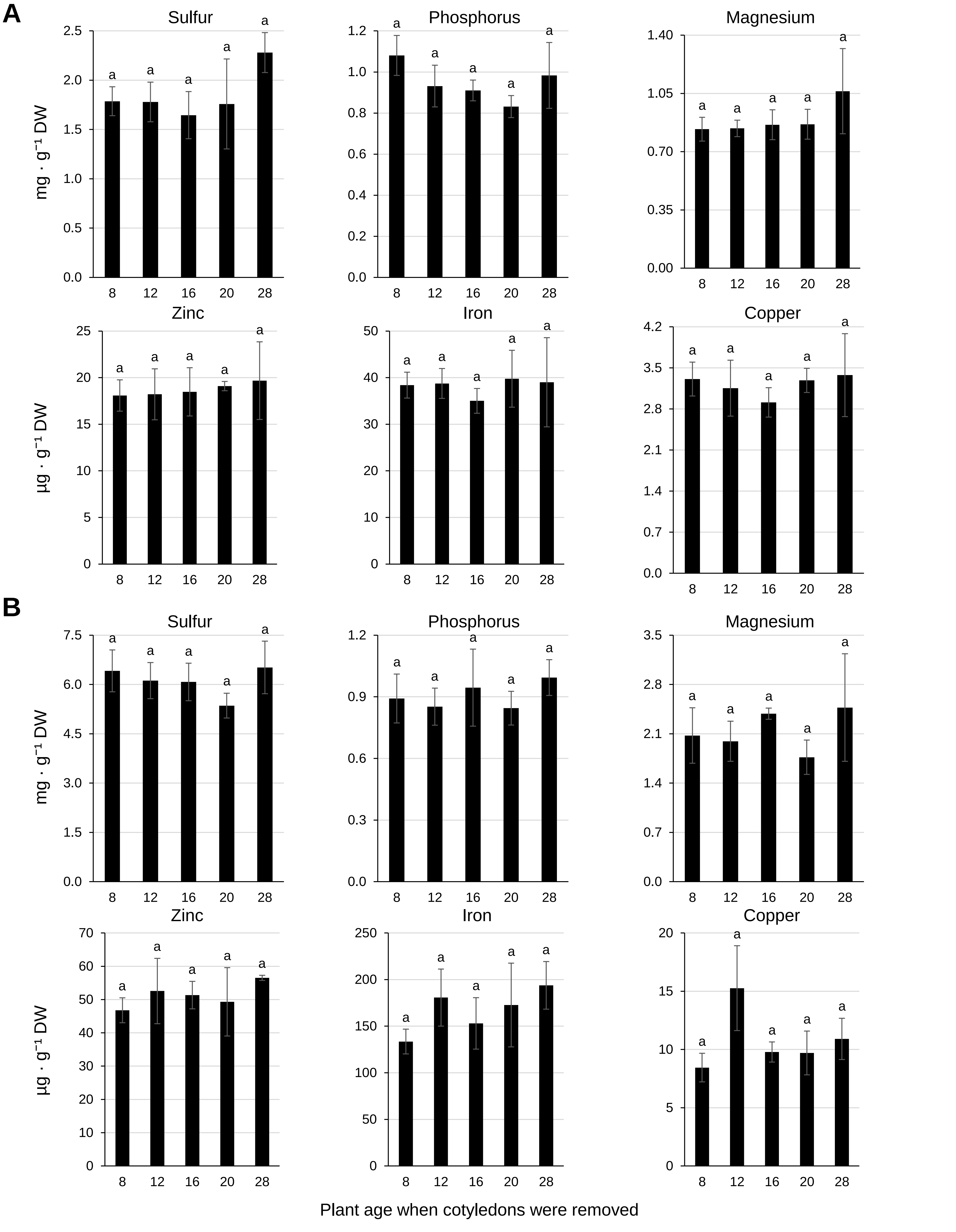

Supplementary Figure S5: Mineral contents of shoots and roots at day 28. (**A**) Shoot. (**B**) Root. Data presented are means ± SD (n = 3). Means were compared using one-way ANOVA followed by Tukey's HSD test. Letter-based significance grouping was used at a significance level of $p < 0.05$; means sharing the same letter are not significantly different. Complete dataset is provided as Supplementary dataset S1.

Nitrogen saving strategies after removal of cotyledons:

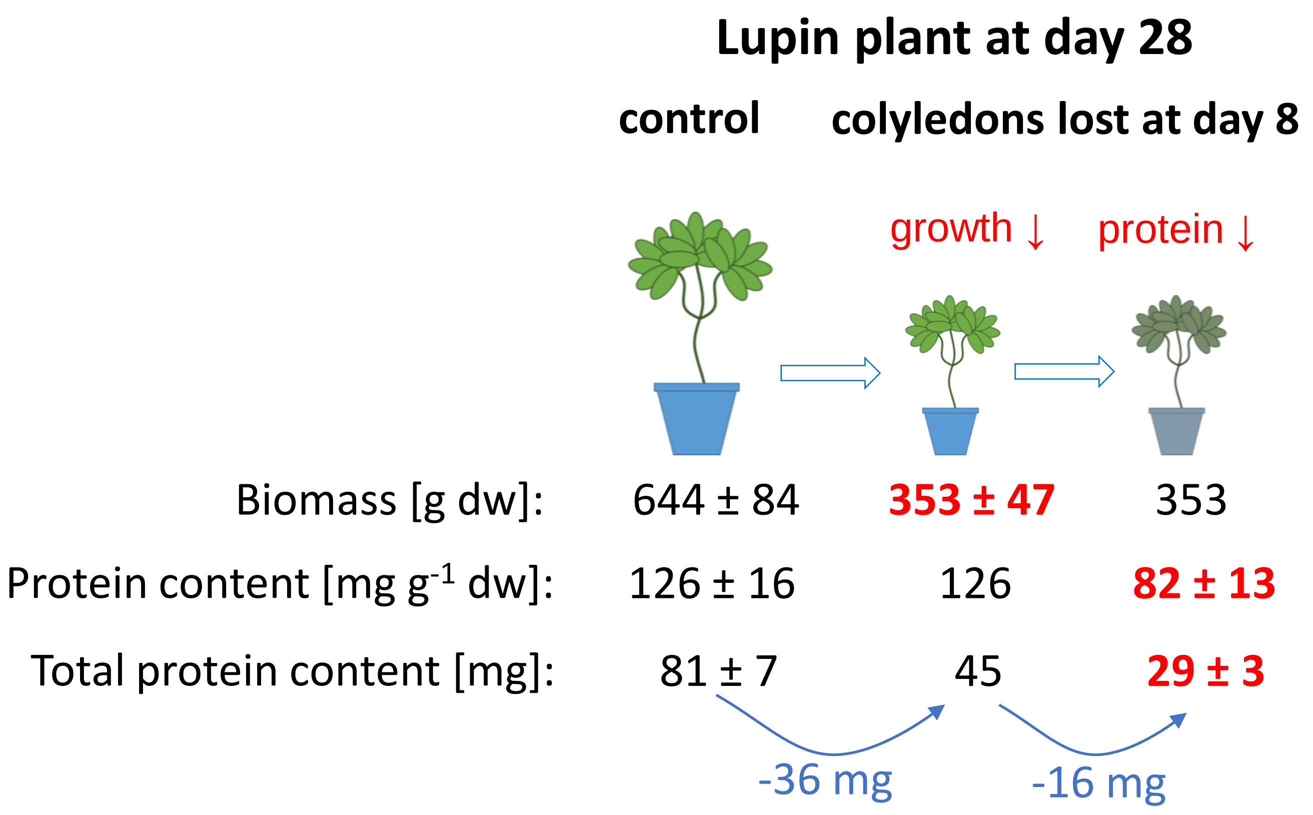

1. Reducing **plant biomass** from 644 to 353 mg dry weight

   ⇨ saves **36 mg protein**

2. Reducing **protein content** from 126 to 82 mg protein g^{-1} dry weight

   ⇨ saves **16 mg protein**

Supplementary Figure S6: Nitrogen saving strategies after removal of the cotyledons: The plant is able to save 15.5 mg protein by reducing its relative protein content by 35 % from 126 ±16 to 82 ± 13 mg · g-1 DW after premature cotyledon loss at day 8. The most effective nitrogen saving strategy, however, is the reduction in plant biomass from 644 ± 84 to 353 ± 47 mg dry weight that, based on the mean tissue protein content of the control plant, would save 36.7 mg protein.
